# β2-adrenergic signals downregulate the innate immune response and reduce host resistance to viral infection

**DOI:** 10.1084/jem.20190554

**Published:** 2020-02-11

**Authors:** Elisabeth Wieduwild, Mathilde J. Girard-Madoux, Linda Quatrini, Caroline Laprie, Lionel Chasson, Rafaëlle Rossignol, Claire Bernat, Sophie Guia, Sophie Ugolini

**Affiliations:** 1 Aix Marseille University, Centre National de la Recherche Scientifique, Institut National de la Santé et de la Recherche Médicale, Centre d’Immunologie de Marseille-Luminy, Marseille, France; 2 Department of Immunology, Istituto di Ricovero e Cura a Carattere Scientifico Bambino Gesù Children’s Hospital, Rome, Italy

## Abstract

In humans, psychological stress has been associated with a higher risk of infectious illness. However, the mechanisms by which the stress pathway interferes with host response to pathogens remain unclear. We demonstrate here a role for the β2-adrenergic receptor (β2-AR), which binds the stress mediators adrenaline and noradrenaline, in modulating host response to mouse cytomegalovirus (MCMV) infection. Mice treated with a β2-AR agonist were more susceptible to MCMV infection. By contrast, β2-AR deficiency resulted in a better clearance of the virus, less tissue damage, and greater resistance to MCMV. Mechanistically, we found a correlation between higher levels of IFN-γ production by liver natural killer (NK) cells and stronger resistance to MCMV. However, the control of NK cell IFN-γ production was not cell intrinsic, revealing a cell-extrinsic downregulation of the antiviral NK cell response by adrenergic neuroendocrine signals. This pathway reduces host immune defense, suggesting that the blockade of the β2-AR signaling could be used to increase resistance to infectious diseases.

## Teaser

Stress hormones increase host susceptibility to viral infection. β_2_-adrenergic signals in nonhematopoietic cells downmodulate the proinflammatory antiviral natural killer cell response, which is necessary for efficient pathogen elimination. This modulation increases the severity of tissue lesions, decreasing resistance to infection.

## Graphical Abstract

**Figure:**
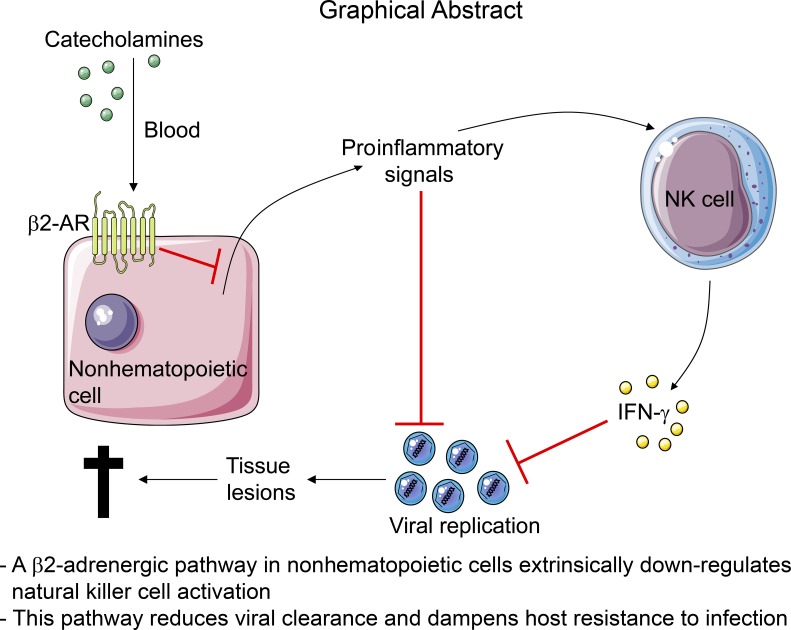

## Introduction

Protection against infection is thought to be ensured principally by the host’s immune system. However, several studies have revealed the importance of neuroimmune regulation in host resistance to infections (Quatrini et al., 2018a; Rankin and Artis, 2018). Receptors for neurohormones, such as glucocorticoids, adrenaline, and noradrenaline, regulate immune cell functions in infectious diseases (Moriyama et al., 2018; Quatrini et al., 2018b; Quatrini et al., 2017). Adrenaline and noradrenaline are produced upon activation of the sympathetic nervous system and transmit signals from the brain to the peripheral tissues. They bind to adrenergic receptors (ARs) expressed by many cell types, including immune cells (Elenkov et al., 2000). Adrenergic signals can have pleiotropic effects. They have been shown to control myeloid cell migration into tissues by controlling adhesion molecule and chemoattractant expression by vascular endothelial cells (Scheiermann et al., 2012). In adaptive lymphocytes, signals mediated by β2-ARs control lymphocyte dynamics by altering the responsiveness of chemoattractant receptors (Nakai et al., 2014). After stroke or cerebral artery occlusion, high levels of sympathetic activity can induce changes in the behavior of invariant natural killer (NK) T cells in the liver or NK cell counts in the spleen (Wong et al., 2011; Liu et al., 2017). The β2-AR pathway is also a cell-intrinsic negative regulator of type 2 innate lymphoid cell (ILC) responses in the intestine, acting through the inhibition of effector function and cell proliferation (Moriyama et al., 2018). However, the role of the β2-AR pathway in viral infections in vivo is poorly understood.

Here, we dissect the role of the β2-AR pathway in controlling early immune responses and resistance to mouse CMV (MCMV). MCMV is commonly used as a model of human CMV infection. The initial cytokine response to MCMV infection includes type 1 IFNs, IL-12, TNF-α, IL-6, and IL-18, which are produced principally by myeloid cells. These proinflammatory cytokines mediate various antiviral effects, including NK cell activation (Biron and Tarrio, 2015). NK cells play a major role in the early innate immune response to MCMV (Lam and Lanier, 2017). Type 1 IFN enhances NK cell–mediated killing, whereas IL-12 induces IFN-γ production by these cells (Biron and Tarrio, 2015). In C57BL/6 mice, NK cells can also be directly activated through recognition of MCMV-infected cells by the activating receptor Ly49H (Dokun et al., 2001; Daniels et al., 2001; Arase et al., 2002; Smith et al., 2002). It has recently been shown that liver-resident ILC1s also confer early host protection against MCMV infection through their IFN-γ production (Weizman et al., 2017).

Here, we investigated the role of the β2-AR pathway in controlling the host response to MCMV. We found that mice treated with a β2-AR agonist were more susceptible to MCMV infection. By contrast, β2-AR–deficient mice (*Adrb2^−/−^* mice) produced higher levels of inflammatory cytokines and were more resistant to MCMV infection than their littermate controls. This phenotype was associated with a better clearance of the virus and less tissue damage in the spleen of infected mice. We analyzed the underlying regulatory mechanisms using genetic dissection, including conditional β2-AR depletion in lymphoid or myeloid cell subsets and bone marrow (BM) chimera experiments.

## Results and discussion

### The β2-AR pathway regulates host resistance to MCMV infection

**Figure 1.**
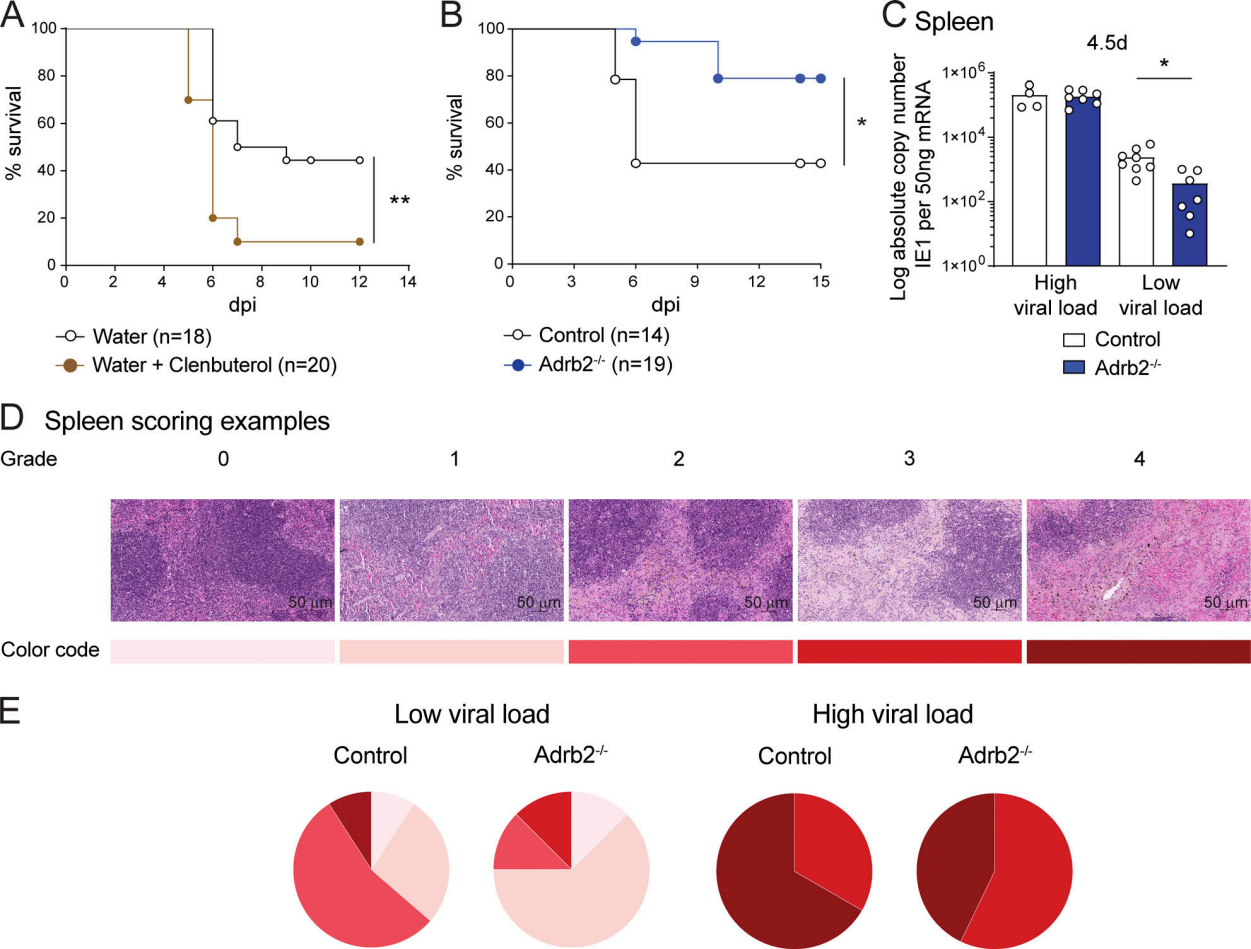
**β2-AR signaling regulates viral clearance and resistance to MCMV infection.** **(A)** Survival rate of WT mice infected with MCMV at LD_50_. Mice were treated (filled brown circles) or not (empty black circles) with Clenbuterol in drinking water during 7 d before infection and throughout the experiment (pool of two independent experiments; Mantel-Cox test, *, P < 0.05). **(B)** Survival rate of *Adrb2^−/−^* mice (filled blue circles) and control *Adrb2^+/+^* littermates (empty black circles) after infection with MCMV at LD_50_ (pool of three independent experiments; Mantel-Cox test, *, P < 0.05). **(C)** Viral titers in the spleens of *Adrb2^−/−^* mice (filled blue bars) and control *Adrb2^+/+^* littermates (empty bars) at 4.5 d pi. Mice were grouped according to high (left) and low (right) viral loads (pool of two independent experiments; each point represents one mouse; unpaired *t* test, *, P < 0.05). **(D)** H&E staining of spleen sections after MCMV infection at LD_50_. Histopathological lesions were scored from grade 0 to 4, and a color code was attributed to each score. Scale bars = 50 µm. **(E)** Histopathological analysis of the spleens of infected *Adrb2^−/−^* and control *Adrb2^+/+^* littermates 4.5 d pi with MCMV at LD_50_. Scoring is based on the grading and color code shown in D. The mice were divided into two groups according to their viral loads as shown in B. The frequency of mice with a given pathological score is shown for each group of mice (pool of two independent experiments; *n *= 12–14 per group).

Psychological distress, which is associated with the production of adrenaline and noradrenaline, has been linked to a higher risk of developing acute infectious diseases (Cohen et al., 1991; Glaser and Kiecolt-Glaser, 2005; Irwin and Cole, 2011). We assessed the potential contribution of the β2-AR pathway to this process in a mouse model of acute MCMV infection. We first treated WT C57BL/6J mice with the β2-AR agonist Clenbuterol for 7 d before and during the course of MCMV infection. WT mice treated with Clenbuterol in drinking water were more susceptible to MCMV infection than their untreated littermates (survival rate of 10% vs. 50%, respectively; Fig. 1 A). We investigated the importance of the β2-AR pathway in a more physiological setting without the addition of exogenous stress hormones by comparing host resistance to MCMV in β2-AR–deficient (*Adrb2^−/−^*) and control (*Adrb2^+/+^*) littermates infected with a LD_50_. *Adrb2^−/−^* mice were significantly more resistant to MCMV than control *Adrb2^+/+^* mice (survival rate of 80% vs. 40%, respectively; Fig. 1 B). These data demonstrate that the β2-AR pathway has a deleterious effect on host resistance to acute MCMV infection.

**Figure S1.**
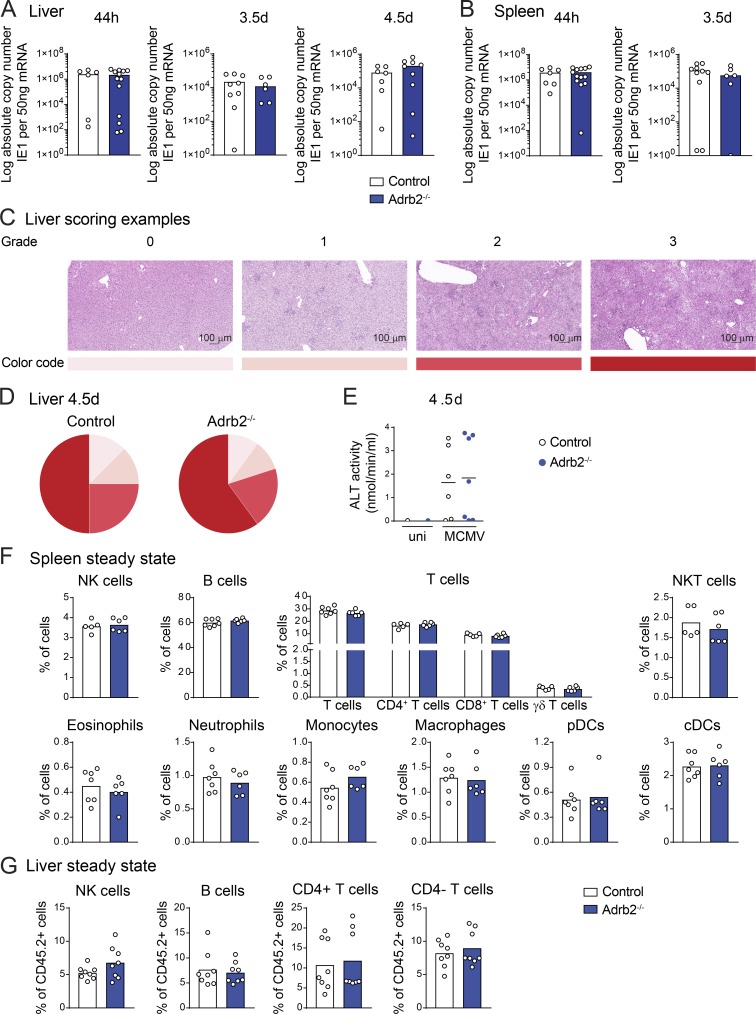
**Viral clearance and tissue damage in the liver of *Adrb2^−/−^* mice after MCMV infection.** **(A)** Viral titer in the liver of *Adrb2^−/−^* mice (filled blue bars) and control *Adrb2^+/+^* littermates (empty bars) at 44 h, 3.5 d, and 4.5 d pi (pool of two independent experiments per time point; each point represents one mouse). **(B)** Viral titer in the spleen of *Adrb2^−/−^* mice (filled blue bars) and control *Adrb2^+/+^* littermates (empty bars) at 44 h and 3.5 d (pool of two independent experiments per time point; each point represents one mouse). **(C)** H&E staining of liver sections after MCMV infection at LD_50_. Shown are examples for the grading used to determine tissue damage and the color code. Scale bars = 100 µm. **(D)** Histological inflammatory scores of livers from *Adrb2^−/−^* mice and control *Adrb2^+/+^* littermates 4.5 d pi with MCMV at LD_50_. Scoring was based on the grading shown in C. **(E**) ALT activity in the serum of *Adrb2^−/−^* mice (filled blue circles) and control *Adrb2^+/+^* littermates (empty circles) at 4.5 d pi (one experiment). **(F)** Frequency of NK, B, T, and NK T cells, eosinophils, neutrophils, monocytes, macrophages, pDCs, and classical DCs (cDCs) in the spleen of *Adrb2^−/−^* mice (filled blue bars) and control littermates (empty bars) at steady state (pool of two independent experiments; each point represents one mouse). **(G)** Frequency of NK, B, and T cells among total CD45.2^+^ cells in the liver of *Adrb2^−/−^* mice (filled blue bars) and control littermates (empty bars) at steady state (pool of two independent experiments; each point represents one mouse). uni, uninfected.

We investigated the mechanisms involved by measuring the viral load over the course of MCMV infection in the spleen and the liver, the principal organs in which the virus replicates (Biron and Tarrio, 2015). Liver viral loads were similar in the two genotypes at 44 h, 3.5 d, and 4.5 d post-infection (pi; Fig. S1 A). In addition, a histological analysis of tissue damage 4.5 d pi revealed no difference between the livers of *Adrb2^−/−^* and control mice (Fig. S1, C and D). Consistent with this result, an analysis of serum alanine aminotransferase (ALT) activity levels, an indicator of liver disease, revealed no difference between β2-AR–deficient and WT animals (Fig. S1 E). The viral loads were also similar in the spleens of *Adrb2^−/−^* and control mice 44 h and 3.5 d pi (Fig. S1 B). However, 4.5 d pi, just before the first animals began to succumb to the disease (Fig. 1 B), we were able to identify two groups of mice: those with high viral loads (>10^4^ copies of the viral genome/50 ng mRNA) and mice with lower viral loads (<10^4^ copies of the viral genome/50 ng mRNA; Fig. 1 C). This dichotomy is consistent with the use of an infectious dose close to the LD_50_ to generate infections from which only some of the mice would be expected to recover (Fig. 1 B). In the group of mice with lower viral loads, viral clearance in the spleen was more efficient in *Adrb2^−/−^* than in control *Adrb2^+/+^* mice (Fig. 1 C). During MCMV infection, the viral load is correlated with the severity of tissue damage and subsequent host survival (Bukowski et al., 1984). We performed histological analyses on the spleens of these animals to determine whether these differences in viral load could explain the higher survival of mice lacking the β2-AR. The severity of spleen lesions was scored from 0 to 4 (Fig. 1 D). In the group of mice with low viral loads, the control mice had a higher frequency of high-grade lesions, most of the scores obtained being between 2 and 4, whereas *Adrb2^−/−^* mice had scores between 0 and 1 (Fig. 1 E). Collectively, these data suggest that, in response to acute MCMV infection, the β2-AR pathway reduces the ability of mice to clear the virus, increasing the severity of virus-induced lesions in the spleen and reducing host resistance to the disease.

### The β2-AR signaling pathway downregulates the inflammatory cytokine response to MCMV

We then decided to analyze the mechanisms involved in the greater resistance of *Adrb2^−/−^* mice to MCMV infection, by analyzing their immune system at steady state and after infection. We first investigated the role of the β2-AR pathway in hematopoietic development and homeostasis by analyzing the distribution of major immune cell subsets in the spleen, liver, and blood of β2-AR–deficient (*Adrb2^−/−^*) and control (*Adrb2^+/+^*) mice at steady state. *Adrb2^+/+^* and *Adrb2^−/−^* mice had similar numbers of neutrophils, monocytes, and T, B, and NK cells, in the blood (data not shown). *Adrb2^+/+^* and *Adrb2^−/−^* mice also had similar frequencies of NK, B, T, and NK T cells, eosinophils, neutrophils, monocytes, and dendritic cells (DCs) in the spleen, as well as NK, B, and T cells in the liver (Fig. S1, F and G). These results are consistent with previous studies (Sanders et al., 2003) and show that β2-AR deficiency does not impair the development or homeostasis of major immune cell subsets in homeostatic conditions.

**Figure S2.**
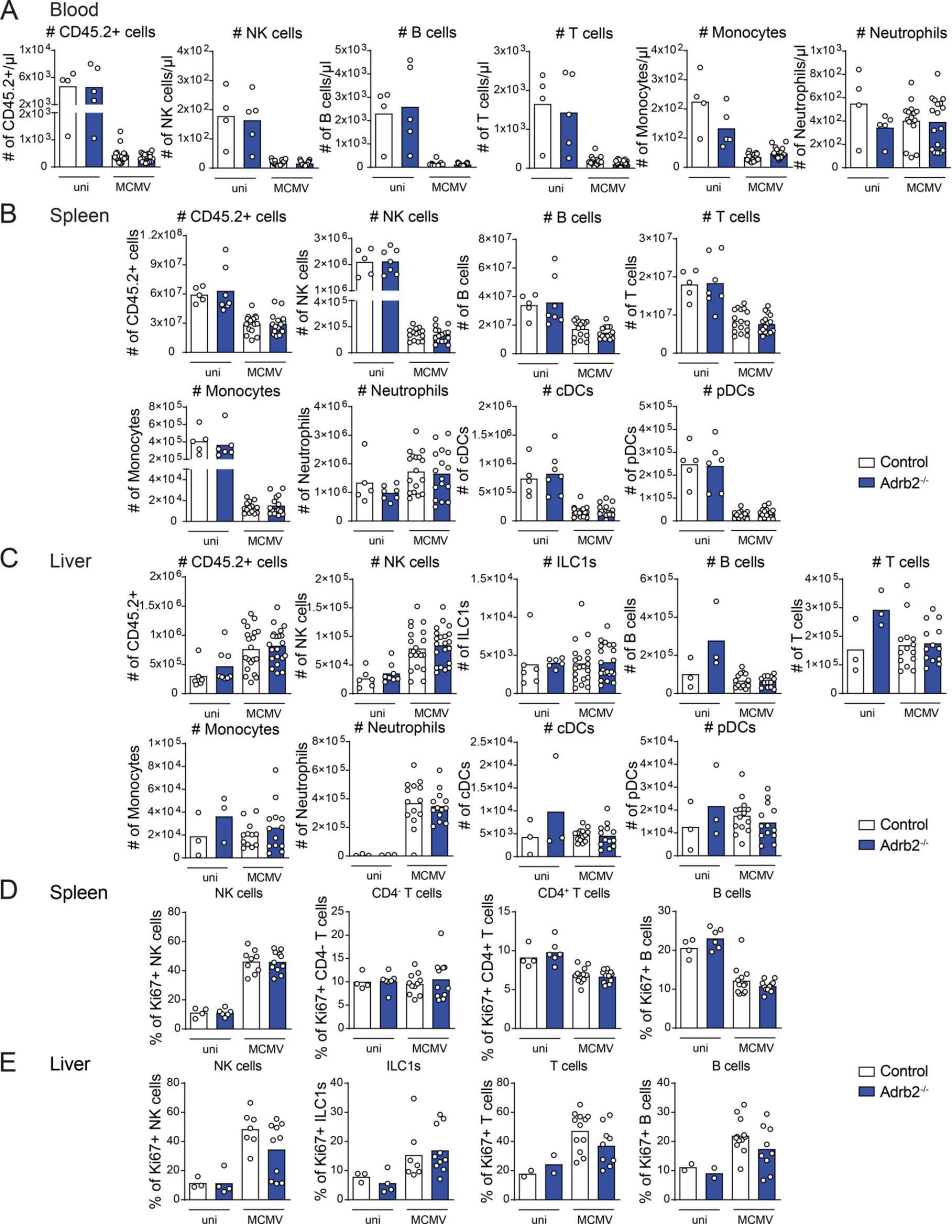
**β2-AR deficiency does not alter the trafficking of major immune cell subsets upon MCMV infection.** **(A–C)** Immune cell subsets were analyzed in the (A) blood, (B) spleen, and (C) liver of *Adrb2^−/−^* mice (filled blue bars) and control littermates (empty bars) at 44 h pi (pool of three independent experiments; each point represents one mouse). **(A)** Total number of CD45.2^+^ cells, B cells (CD45.2^+^, CD19^+^), monocytes (CD45.2^+^, TCRβ^−^, NKp46^−^, CD19^−^, GR-1^+^), neutrophils (CD45.2^+^, GR-1^high^), NK cells (CD45.2^+^, TCRβ^−^, CD19^−^, NKp46^+^, NK1.1^+^) and T cells (CD45.2^+^, CD19^−^ TCRβ^+^). **(B)** Total number of CD45.2^+^ cells, NK cells (CD45.2^+^, TCRβ^−^, CD19^−^, Ly6G^−^, NKp46^+^, NK1.1^+^), B cells (CD45.2^+^, TCRβ^−^, NKp46^−^, Ly6G^−^, CD19^+^), T cells (CD45.2^+^, TCRβ^+^, CD19^−^, NKp46^−^, Ly6G^−^), monocytes (CD45.2^+^, TCRβ^−^, NKp46^−^, Ly6G^−^, CD19^−^, CD11c^−^, CD11b^high^, Ly6C^high^), neutrophils (CD45.2^+^, TCRβ^−^, NKp46^−^, CD19^−^, Ly6G^+^), classical DCs (cDCs; CD45.2^+^, TCRβ^−^, NKp46^−^, CD19^−^, Ly6G^−^, CD11b^low^, CD11c^+^, MHC-II^+^), and pDCs (CD45.2^+^, TCRβ^−^, NKp46^−^, CD19^−^, Ly6G^−^, CD11b^−^, CD11c^+^, Ly6C^+^). **(C)** Total number of CD45.2^+^ cells, ILC1s (CD49a^+^, CD49b^−^), NK cells (CD49a^−^, CD49b^+^), B cells, T cells, monocytes, neutrophils, cDCs, and pDCs. **(D and E)** Frequency of Ki67^+^ cells among total B, T, and NK cells and ILC1s in the spleen (D) and liver (E) of *Adrb2^−/−^* mice (filled blue bars) and control littermates (empty bars) at 44 h pi (pool of two to three independent experiments; each point represents one mouse). uni, uninfected.

**Figure S3.**
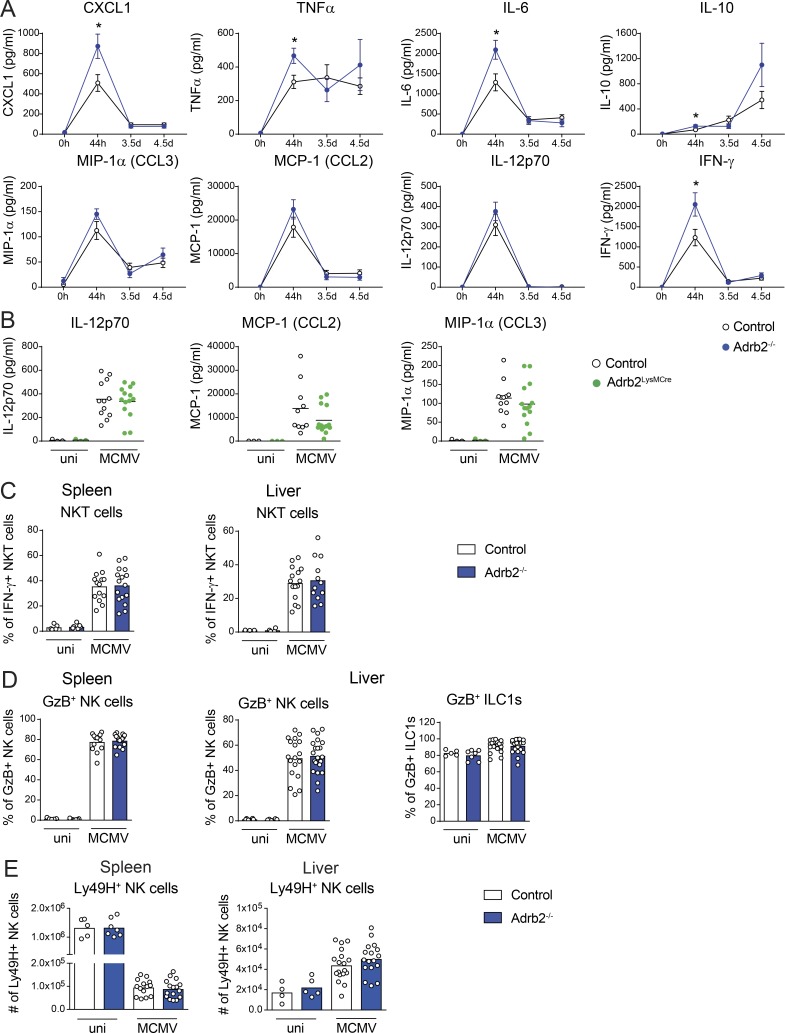
**Inflammatory cytokine response in MCMV-infected *Adrb2^−/−^* and *Adrb2^LysMCre^* mice.** **(A)** Kinetics of CXCL1, TNF-α, IL-6, IL-10, MIP-1α (CCL3), MCP-1 (CCL2), IL-12p70, and IFN-γ production in the serum of *Adrb2^−/−^*mice (filled blue circles) and control littermates (empty circles) after infection with MCMV at LD_50_ (pool of two to three experiments per time point). **(B)** Concentrations of IL-12p70, MCP-1 (CCL2), and MIP-1α (CCL3) in the serum of *Adrb2^LysMCre^* mice (filled green circles) and control littermates (empty circles) 44 h pi (pool of three independent experiments; each point represents one mouse). **(C)** Total number of IFN-γ–producing NK T cells in the liver (left) and spleen (right) of *Adrb2^−/−^* mice (filled blue bars) and control littermates (empty bars) at 44 h pi (pool of three independent experiments; each point represents one mouse). **(D)** Frequency of GzB expressing NK cells in the spleen and liver and frequency of GzB-expressing ILC1s in the liver of *Adrb2^−/−^* mice (filled blue bars) and control littermates (empty bars) at 44 h pi (pool of three independent experiments; each point represents one mouse). **(E)** Total number of Ly49H^+^ NK cells in the spleen (left) and liver (right) of *Adrb2^−/−^* mice (filled blue bars) and control littermates (empty bars) at 44 h pi (pool of three independent experiments; each point represents one mouse). uni, uninfected.

**Figure 2.**
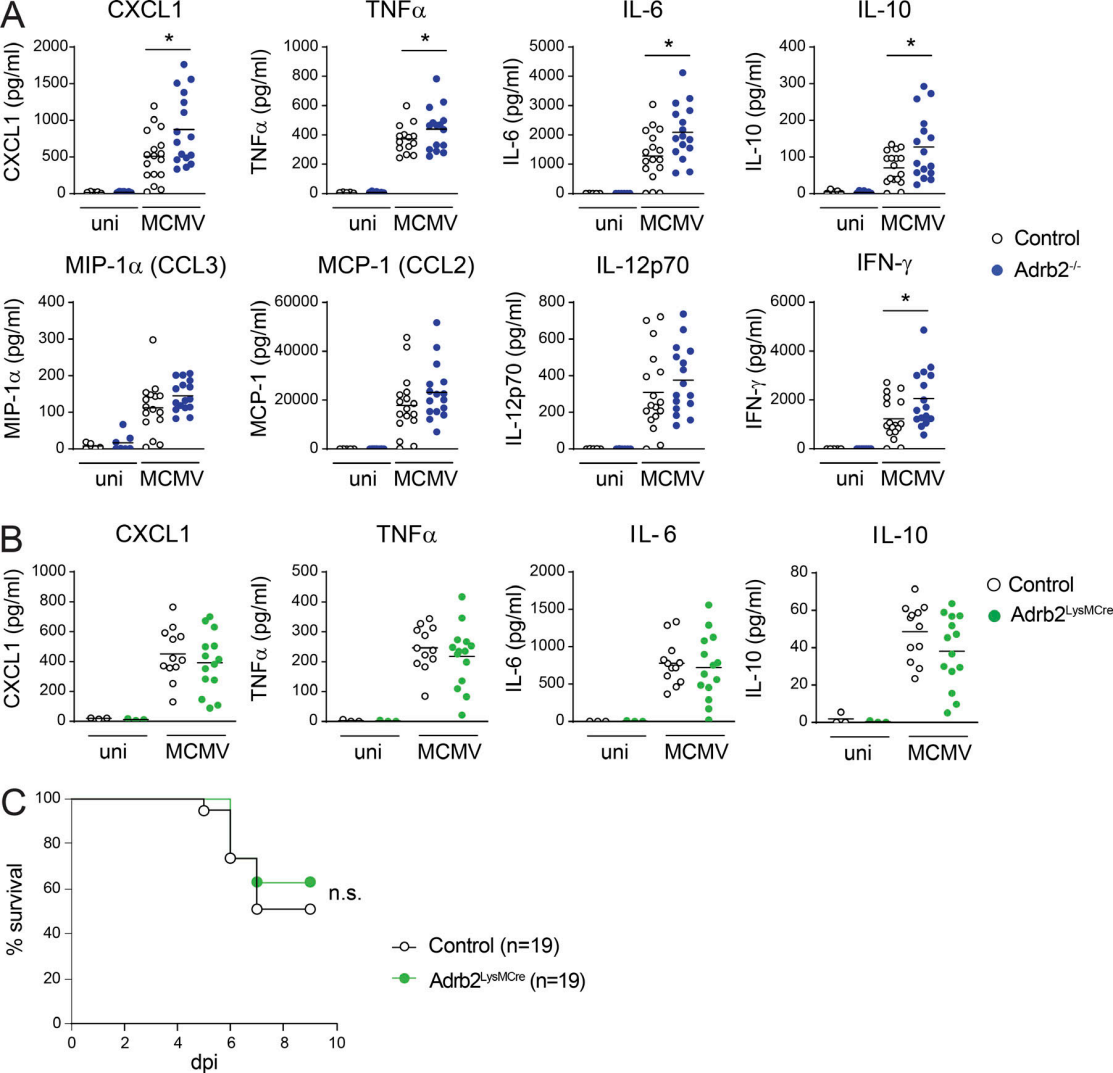
**β2-AR signals downregulate the cytokine and chemokine responses to MCMV.** **(A)** Concentrations of CXCL1, TNF-α, IL-6, IL-10, MIP-1a (CCL3), MCP1 (CCL2), IL-12p70, and IFN-γ in the serum of *Adrb2^−/−^* (filled blue circles) and control *Adrb2^+/+^* littermates (empty circles) at 44 h pi (pool of three independent experiments; each point represents one mouse; unpaired *t* test, *, P < 0.05). **(B)** Concentrations of CXCL1, TNF-α, IL-6, and IL-10 in the serum of *Adrb2^LysMCre^* mice (filled green circles) and controls (empty circles) at 44 h pi (pool of three independent experiments; each point represents one mouse). **(C)** Survival of *Adrb2^LysMCre^* mice (filled green circles) and control littermates (empty circles) after infection with MCMV at LD_50_ (pool of three independent experiments). uni, uninfected mice; n.s., not significant.

We then investigated whether β2-AR signaling regulated the host immune response to MCMV infection. Depending on the cell type and the pathological context, the β2-AR pathway can regulate immune cell trafficking, survival, or proliferation (Wong et al., 2011; Nakai et al., 2014; Liu et al., 2017; Moriyama et al., 2018). We thus analyzed the distribution of immune cells in the spleen, liver, and blood of infected mice. We detected no differences between *Adrb2^−/−^* and *Adrb2^+/+^* control mice in terms of the distribution of the major subsets of immune cells in these tissues upon MCMV infection (Fig. S2, A–C). Moreover, Ki67 expression revealed no difference in the proliferation of B, T, and NK cells and ILC1s between *Adrb2^−/−^* mice and *Adrb2^+/+^* control mice (Fig. S2, D and E). β2-AR signaling is, therefore, not required for the early modulation of immune cell trafficking or proliferation of the main immune cell subsets in the context of MCMV infection. We then investigated whether immune cell functions were regulated by this pathway. We monitored the early cytokine response induced by MCMV infection in these animals at 44 h, 3.5 d, and 4.5 d pi. The levels of CCL3, CCL2, and IL-12p70 increased similarly in the blood of *Adrb2^−/−^* mice and *Adrb2 ^+/+^*control mice, peaking at 44 h pi (Fig. S3 A). By contrast, levels of CXCL1, TNF-α, IL-6, and IFN-γ production were higher in the bloodstream of infected *Adrb2^−/−^* mice than in that of their control *Adrb2^+/+^* littermates 44 h pi (Fig. 2 A and Fig. S3 A). IL-10 levels, which are usually low at 44 h pi, were also slightly higher at this time point (Fig. 2 A and Fig. S3 A).

These data show that the β2-AR signaling pathway is required to control the magnitude of the early innate inflammatory response to MCMV, particularly in terms of the production of CXCL1, TNF-α, IL-6, and IFN-γ.

### β2-AR expression in LysM^+^ myeloid cells is not required for control of the inflammatory cytokine response to MCMV

Early in MCMV infection, TNF-α, IL-6, IL-10, and CXCL1 are produced, principally by myeloid cells (Biron and Tarrio, 2015). Previous in vitro studies suggested a potential role of β-AR pathways in polarizing BM-derived macrophages toward an anti-inflammatory phenotype (Lamkin et al., 2016). We investigated whether TNF-α, IL-6, CXCL1, and IL-10 levels were modified by the intrinsic regulation of the adrenergic pathway in myeloid cell subsets in vivo in the context of MCMV infection. We studied *LysM^Cre/+^Adrb2^flx/flx^* mice (hereafter referred to as *Adrb2^LysMCre^* mice), in which the *Adrb2* gene is selectively deleted in LysM^+^ cells, including neutrophils, macrophages, monocytes, and DC subsets (Abram et al., 2014) and their littermate controls (*LysM^+/+^Adrb2^flx/flx^*). MCMV infection induced similar increases in CXCL1, IL-6, TNF-α, IL-10, IL-12p70, CCL2, and CCL3 levels in the blood of *Adrb2^LysMCre^* and control mice at 44 h pi (Fig. 2 B and Fig. S3 B). The stronger chemokine and cytokine responses to MCMV infection observed in β2-AR–deficient mice (Fig. 2 A) are not, therefore, due to an intrinsic regulation of LysM^+^ myeloid cell functions by β2-AR. In addition, resistance to MCMV infection was similar in *Adrb2^LysMCre^* and control mice (Fig. 2 C), showing that β2-AR signals in LysM^+^ cells are not required for the modulation of host resistance to infection.

### β2-AR controls IFN-γ production by NK cells in an organ-specific manner

**Figure 3.**
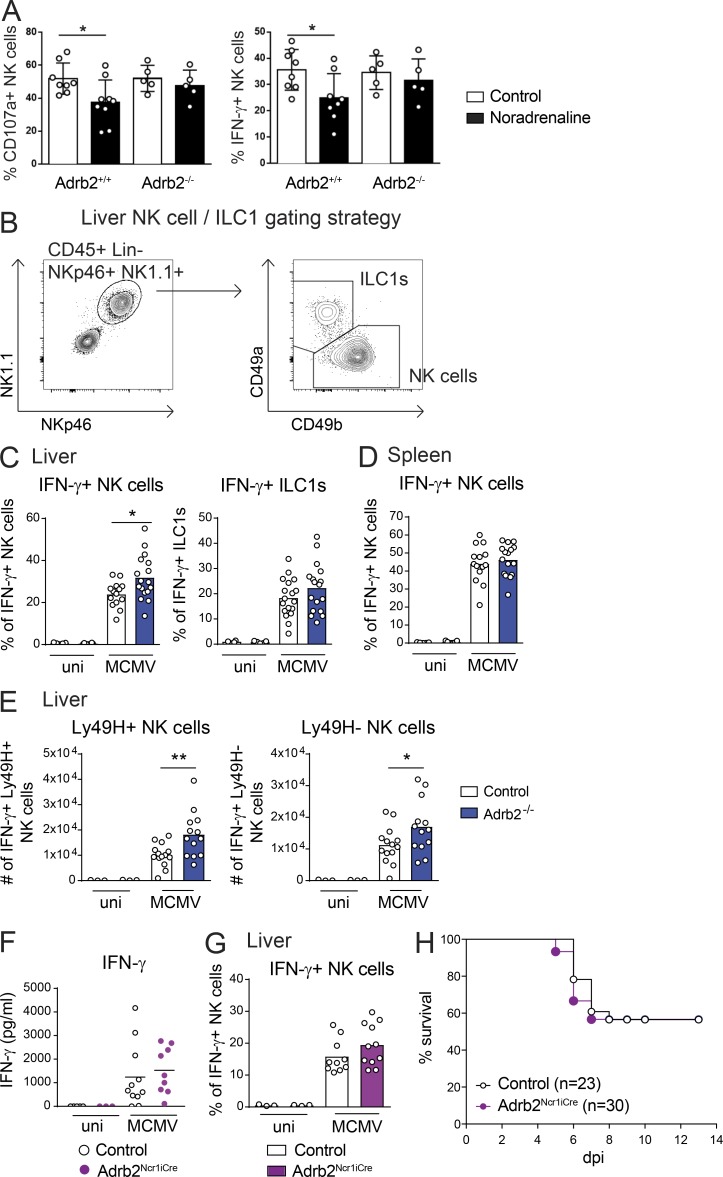
**β2-AR signals regulate IFN-γ production by liver NK cells.** **(A)** Splenocytes from *Adrb2^+/+^* and *Adrb2^−/−^* littermates were stimulated in vitro with anti-NK1.1 mAb-coated plates in the presence (filled black bars) of noradrenaline (10 µM) or diluent (control, empty bars). The frequency of CD107a (left) and IFN-γ–producing NK cells (right) 4 h after stimulation is shown (each point represents one mouse; means ± SEM are shown; unpaired *t* test, *, P < 0.05). **(B)** Gating strategy used to identify NK cells (CD45^+^, CD3^−^, CD8^−^, CD19^−^, Ly6G^−^, NKp46^+^, NK1.1^+^, CD49a^−^, CD49b^+^) and ILC1s (CD45^+^, CD3^−^, CD8^−^, CD19^−^, Ly6G^−^, NKp46^+^, NK1.1^+^, CD49a^+^, CD49b^−^) in the liver. **(C and D)** Frequency of IFN-γ–producing NK cells and ILC1s in the liver (C) and spleen (D) of *Adrb2^−/−^* mice (filled blue bars) and control *Adrb2^+/+^* littermates (empty bars) at 44 h pi (pool of three independent experiments; each point represents one mouse; unpaired *t* test, *, P < 0.05). **(E)** Frequency of IFN-γ–producing Ly49H^+^ (left) and Ly49H^−^ (right) NK cells in the liver of *Adrb2^−/−^* mice (filled blue bars) and control *Adrb2^+/+^* littermates (empty bars) at 44 h pi (pool of three independent experiments; each point represents one mouse; unpaired *t* test, *, P < 0.05; **, P < 0.005). **(F)** Concentration of IFN-γ in the serum of *Adrb2^Ncr1iCre^* mice (filled purple circles) and control littermates (empty circles) at 44 h pi (pool of three independent experiments; each point represents one mouse). **(G)** Frequency of IFN-γ–producing NK cells in the liver of *Adrb2^Ncr1iCre^* mice (filled purple bars) and control littermates (empty bars) at 44 h pi (pool of three independent experiments; each point represents one mouse). **(H)** Survival rate of *Adrb2^Ncr1iCre^* mice (filled purple circles) and control littermates (empty circles) after infection with MCMV at LD_50_ (pool of four independent experiments). uni, uninfected.

NK cells and liver-resident ILC1s play a major role in the response to MCMV infection through their effector functions, which include cytotoxicity and early IFN-γ production (Vivier et al., 2008; Biron and Tarrio, 2015; Weizman et al., 2017). IFN-γ was among the cytokines upregulated in *Adrb2^−/−^* mice relative to their *Adrb2^+/+^* littermates at 44 h pi (Fig. 2 A). Transcriptomic analysis showed that *Adrb2* was expressed in NK cells and ILC1s, both at steady state and 1 d after MCMV infection (see Immunological Genome Project database: http://www.immgen.org/databrowser). Moreover, in this issue, Diaz-Salazar et al. found that *Adrb2* expression in NK cells was upregulated after MCMV infection and revealed that NK cells localize near splenic adrenergic neurons during infection. Previous studies have suggested that signaling via β-AR can regulate both the trafficking and activity of NK cells (Kradin et al., 2001; Pedersen et al., 2016; De Lorenzo et al., 2015; Tarr et al., 2012). However, most of these studies used a pharmacological blockade of the adrenergic pathway, making it difficult to determine whether the effects observed were cell intrinsic or extrinsic. We assessed the functionality of β2-AR in NK cells by studying the effect of noradrenaline on NK cell activation in vitro. We found that noradrenaline inhibited the NK cell degranulation (CD107a expression) and IFN-γ production induced by stimulation with plate-bound anti-NK1.1 antibodies (Fig. 3 A). This inhibition was strictly β2-AR dependent, as it was not observed when NK cells were isolated from *Adrb2^−/−^* mice. We then investigated the in vivo role of this pathway in the context of MCMV infection. The cellular source of the higher levels of IFN-γ production observed in *Adrb2^−/−^* mice was investigated by intracellular staining on innate lymphocytes from infected mice. The frequency of IFN-γ–producing NK cells in the liver was higher in *Adrb2^−/−^* mice (32%) than in their *Adrb2^+/+^* littermates (24%; Fig. 3 C). This regulation of IFN-γ production by the β2-AR pathway was both cell type and organ specific, as it was not observed for liver ILC1s, liver NK T cells, or spleen NK cells (Fig. 3, C and D and Fig. S3 C). MCMV infection also induced a similar increase in granzyme B (GzB) levels in NK cells and ILC1s from the spleen and liver of *Adrb2^−/−^* mice and control *Adrb2^+/+^* littermates (Fig. S3, C and D), suggesting that β2-AR does not regulate the cytotoxic functions of NK cells.

In C57BL/6J mice, host protection against MCMV involves recognition of the virus-encoded glycoprotein m157 by the Ly49H molecule, which is expressed on the surface of a subset of NK cells (Dokun et al., 2001; Daniels et al., 2001; Arase et al., 2002; Smith et al., 2002). We analyzed the role of the β2-AR pathway in the activation program of this subset. The Ly49H^+^ NK cell counts were similar in the spleen and liver of *Adrb2^−/−^* and control *Adrb2^+/+^* littermates at 44 h after MCMV infection (Fig. S3 E). Moreover, the higher frequency of IFN-γ–producing NK cells in the liver of *Adrb2^−/−^* mice at 44 h pi involved both the Ly49H-positive and -negative subsets (Fig. 3 E). These data show that β2-AR signaling does not selectively affect m157-Ly49H–mediated NK cell activation during the early stages of MCMV infection. However, in adoptive transfer experiments, Diaz-Salazar et al. (2020) demonstrated that this pathway could be important to elicit robust adaptive NK cell responses at later time points.

The differential effect of β2-AR on the control of IFN-γ production in spleen and liver NK cells raised questions concerning the intrinsic or extrinsic nature of this regulation. We analyzed the MCMV response in *Ncr1^Cre/+^Adrb2^flx/flx^* mice (hereafter referred to as *Adrb2^Ncr1iCre^* mice), in which the *Adrb2* gene is deleted selectively in NCR1^+^ cells, including NK cells and ILC1s (Narni-Mancinelli et al., 2011). By contrast to the phenotype observed in *Adrb2^−/−^* mice, no differences in serum IFN-γ levels or the frequency of IFN-γ–producing NK cells were observed between *Adrb2^Ncr1iCre^* mice and their littermate controls (*Ncr1^Cre/+^Adrb2^+/+^*; Fig. 3, F and G). Furthermore, the survival rates were similar for infected *Adrb2^Ncr1iCre^* and control mice (Fig. 3 H). Thus, upon MCMV infection, β2-AR selectively downregulates IFN-γ production by liver NK cells but not by spleen NK cells. This organ-specific regulation is NK cell extrinsic and results in a systemic decrease in IFN-γ levels in the serum, potentially affecting host resistance to infection.

### β2-AR signaling in hematopoietic and nonhematopoietic cells differentially regulates the cytokine response to MCMV

**Figure 4.**
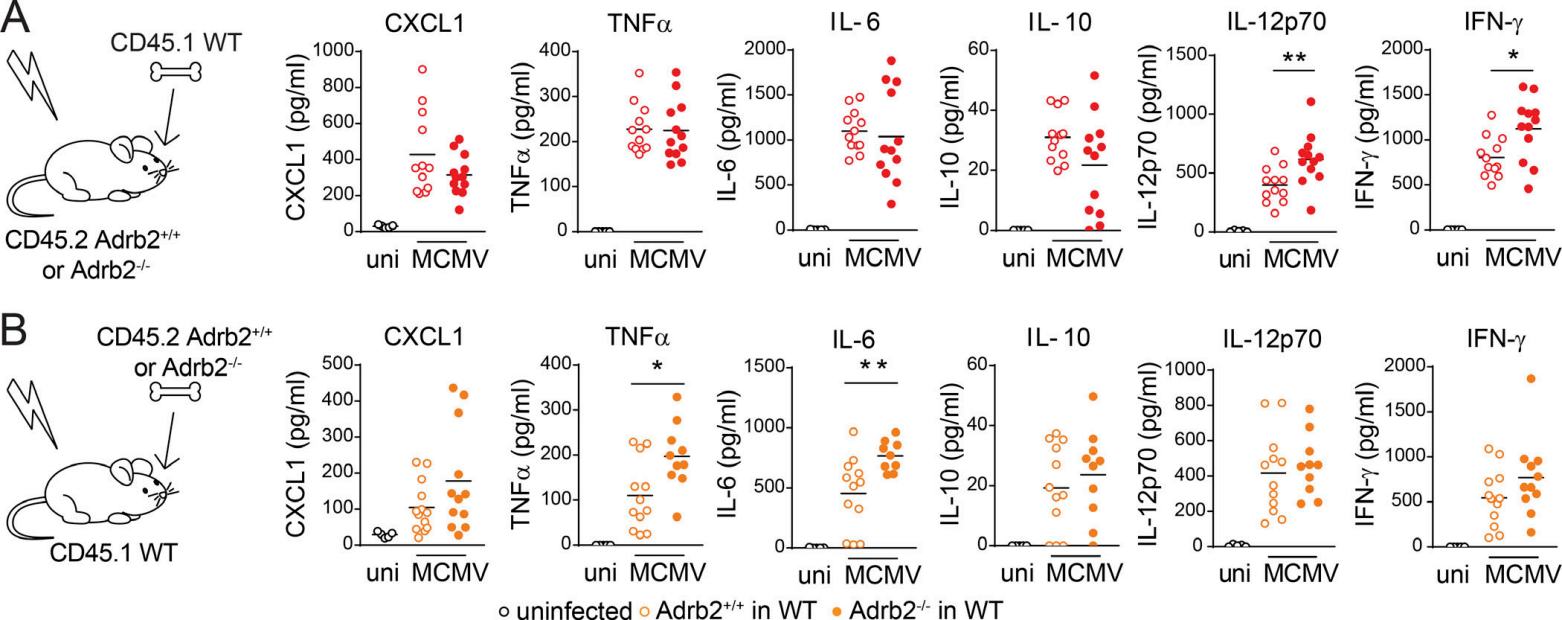
**Differential regulation of the inflammatory cytokine response by β2-AR signaling in hematopoietic and nonhematopoietic cells.** **(A)** Concentration of CXCL1, TNF-α, IL-6, IL-10, IL-12p70, and IFN-γ in the serum of (WT→*Adrb2^−/−^*) BM-chimeras (filled red circles) and (WT→*Adrb2^+/+^*) BM-chimeras (empty red circles) at 44 h pi (pool of three independent experiments; each point represents one mouse; unpaired *t* test, *, P < 0.05; **, P < 0.005). **(B)** Concentration of CXCL1, TNF-α, IL-6, IL-10, IL-12p70, and IFN-γ in the serum of (*Adrb2^−/−^*→WT) BM-chimeras (filled orange circles) and (*Adrb^+/+^*→WT) BM-chimeras (empty orange circles) at 44 h pi (pool of three independent experiments; each point represents one mouse; unpaired *t* test, *, P < 0.05; **, P < 0.005). Data obtained for uninfected mice (uni) are shown.

We found that β2-AR signaling downregulated the early cytokine and chemokine response to MCMV (Fig. 2 A). However, the conditional deletion of *Adrb2* in LysM^+^ or NCR1^+^ cells was not sufficient to modulate the production of IL-6, TNF-α, CXCL1, IFN-γ, or IL-10 or to affect mouse susceptibility to MCMV infection (Fig. 2 B and Fig. 3, F–H). The early host response to MCMV involves both hematopoietic and nonhematopoietic cells (Loewendorf and Benedict, 2010). To further investigate the regulatory mechanisms underlying the enhanced immune response and resistance of MCMV-infected *Adrb2^−/−^* mice, we performed BM chimera experiments. First, CD45.1-WT BM cells were transplanted into lethally irradiated CD45.2-*Adrb2^+/+^* or -*Adrb2^−/−^* recipients from the same litter. Chimeric (WT→*Adrb2^+/+^*) and (WT→*Adrb2^−/−^*) mice were infected, and their production of chemokines and cytokines was monitored by analyzing blood samples collected 44 h after MCMV infection. The production of CXCL1, TNF-α, IL-10, and IL-6 increased similarly in both types of chimera, indicating that β2-AR expression in nonhematopoietic radioresistant cells is not sufficient to modulate the expression of these cytokines and chemokines (Fig. 4 A). By contrast, a defect of β2-AR signaling in nonhematopoietic radioresistant cells was sufficient to enhance the systemic production of IL-12p70 and IFN-γ (Fig. 4 A). We then generated chimeras in which BM cells from CD45.2-*Adrb2^+/+^* or -*Adrb2^−/−^* littermates were transplanted into irradiated CD45.1-WT hosts. After MCMV infection, the levels of TNF-α and IL-6, but not of IL-10, CXCL1, IL-12p70, or IFN-γ, were higher in the blood of mice with BM-derived cells lacking β2-AR (*Adrb2^−/−^*→WT mice) than in control (*Adrb2^+/+^*→WT) mice (Fig. 4 B). These BM chimera experiments show that the deletion of β2-AR in BM-derived hematopoietic cells is necessary and sufficient to upregulate TNF-α and IL-6 production upon MCMV infection. We found no difference in TNF-α and IL-6 levels in the *Adrb2^LysMCre^* mice (Fig. 2 B), demonstrating that the regulation of cytokine production by β2-AR is not intrinsic to LysM^+^ cells. *LysM-cre* promotes significant deletion in macrophages and neutrophils but not in DC subsets, including plasmacytoid DCs (pDCs; Abram et al., 2014). One of the earliest sources of TNF-α during MCMV infection is the pDCs (Biron and Tarrio 2015). A role of the β2-AR on LysM^−^ DCs might, therefore, account for the higher levels of IL-6 and TNF-α production in (*Adrb2^−/−^*→WT) chimeras and in *Adrb2^−/−^* mice. By contrast, the regulation of CXCL1 and IL-10 required the expression of β2-AR in both hematopoietic and nonhematopoietic cells (Fig. 2 A and Fig. 4, A and B). We also found that the regulation of IFN-γ levels required the expression of β2-AR on nonhematopoietic cells (Figs. 2 A and 4, A and B). In BM-chimera settings, the stronger IFN-γ response was associated with higher levels of IL-12p70 production. IL-12p70 was not significantly upregulated in conditions of complete *Adrb2* knockout, although there was a trend in this direction (Fig. 2 A). Therefore, we cannot exclude a role of IL-12 in the upregulation of IFN-γ levels in the absence of β2-AR regulation. Nevertheless, our data suggest that the β2-AR pathway modulates the expression/production of other, still unidentified, factors in nonhematopoietic cells that are involved in upregulating the IFN-γ response.

Collectively, these data show that β2-AR signals downregulate the early inflammatory cytokine response to MCMV through a combination of pleiotropic effects in both BM-derived hematopoietic cells and nonhematopoietic cells.

### The higher resistance of β2-AR–deficient mice to MCMV is associated with higher IFN-γ levels and is partially NK cell dependent

**Figure 5.**
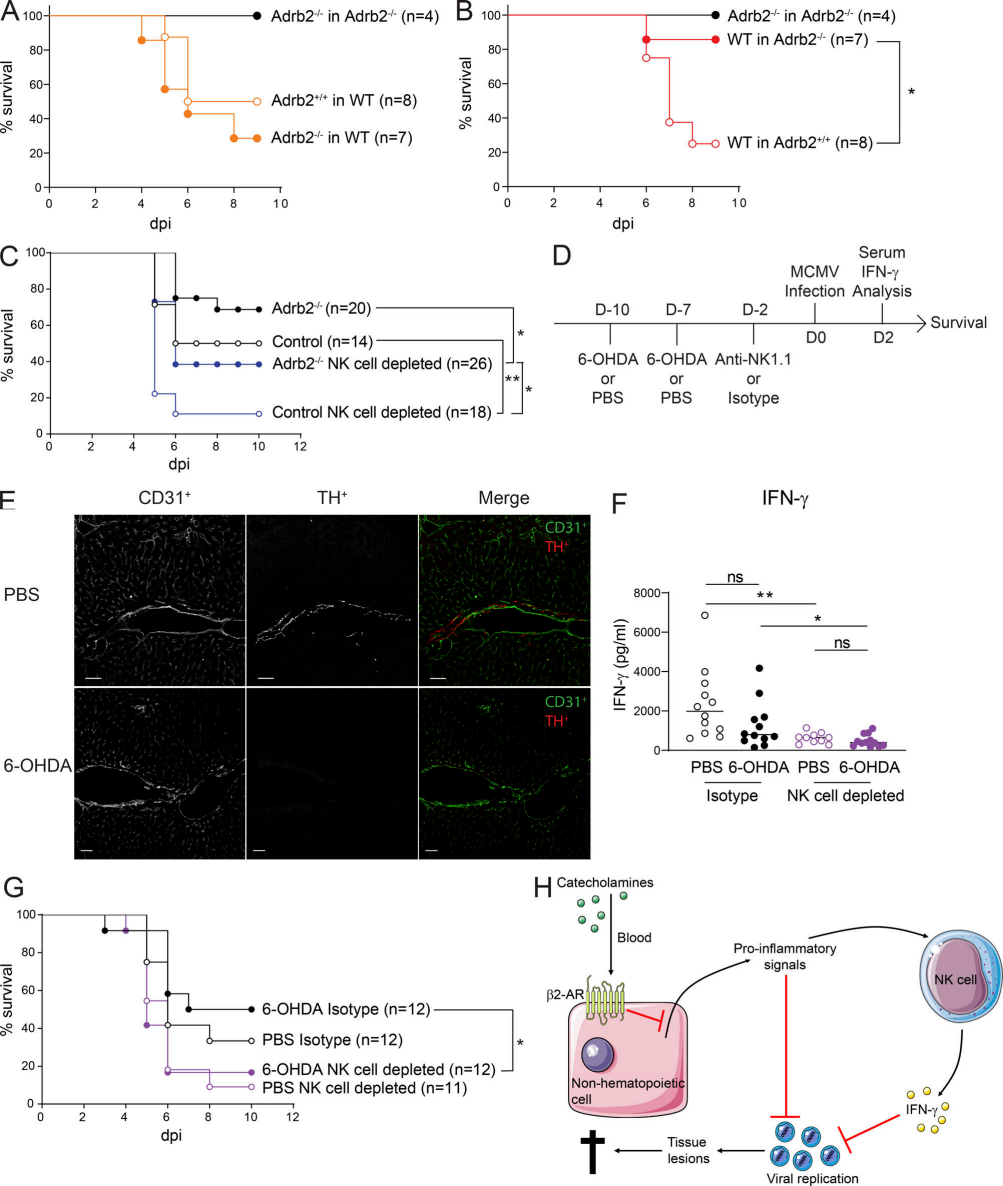
**β2-AR signaling in nonhematopoietic cells regulates resistance to MCMV infection independently of catecholaminergic innervation.** **(A)** Survival rate of (*Adrb2^−/−^*→WT; filled orange circles), (*Adrb2^+/+^*→WT; empty orange circles), and (*Adrb2^−/−^*→*Adrb2^−/−^*; filled black circles) BM chimeras after MCMV infection at LD_50_ (pool of one to two experiments). **(B)** Survival rate of (WT→*Adrb2^−/−^*; filled red circles), (WT→*Adrb2^+/+^*; empty red circles), and (*Adrb2^−/−^*→*Adrb2^−/−^*; filled black circles) BM chimeras after MCMV infection at LD_50_ (pool of one to two experiments; Mantel-Cox test, *, P < 0.05). **(C)** Survival rate of *Adrb2^−/−^* mice (filled circles) and control littermates (empty circles) with (blue circles) or without (black circles) NK cell depletion with anti-NK1.1 mAb treatment 2 d before MCMV infection (pool of three independent experiments; Mantel-Cox test, *, P < 0.05; **, P < 0.005). **(D)** Experimental design for the experiments presented in E, F, and G. **(E)** Immunofluorescence analysis of liver sections from mice treated with 6-OHDA or PBS as control. Endothelial cells from blood vessels were stained with anti-CD31 antibody (left, white staining; right, green staining in overlaid images). TH^+^ nerves were stained with anti-TH antibodies (middle, white staining; right, red staining in overlaid images; scale bars = 50 µm). **(F)** Serum IFN-γ concentrations of 6-OHDA–treated mice (filled circles) and control mice (empty circles) with NK1.1 cell depletion (purple circles) or without NK1.1 cell depletion (black circles) after infection with MCMV at LD_50_ (pool of two independent experiments; each point represents one mouse; Mann-Whitney *U* test, *, P < 0.05; **, P < 0.05). **(G)** Survival rate of 6-OHDA treated mice (filled circles) and control mice (empty circles) with NK1.1 cell depletion (purple circles) or without NK1.1 cell depletion (black circles) after infection with MCMV at LD_50_ (pool of two independent experiments; Mantel-Cox test, *, P < 0.05). **(H)** Model: β2-AR signaling induced by catecholamines produced systemically in the blood circulation act on nonhematopoietic cells to modulate proinflammatory signals. This modulation downregulates the NK cell IFN-γ response, which is necessary for efficient viral clearance. This pathway reduces the control of viral replication, increases the severity of spleen lesions, and dampens host resistance to infection. uni, uninfected.

We investigated the mechanism by which the β2-AR pathway regulates host susceptibility to MCMV by monitoring mouse survival in BM-chimera animals displaying differential regulation of the inflammatory cytokines TNF-α, IL-6, IL-12p70, and IFN-γ (Fig. 4). The upregulation of IL-6 and TNF-α levels in (*Adrb2^−/−^*→WT) chimeras was not associated with greater resistance to MCMV infection, as the survival of these animals was similar to that of (*Adrb2^+/+^*→WT) mice (Fig. 5 A). By contrast, the deletion of β2-AR in nonhematopoietic recipient cells, enhancing the production of IL-12p70 and IFN-γ (Fig. 4 A), was sufficient to increase host resistance to MCMV infection, as the survival of (WT→*Adrb2^−/−^*) chimeras (82%) was greater than that of the corresponding (WT→*Adrb2^+/+^*) controls (20%; Fig. 5 B). Together, these data show that the higher levels of IFN-γ production in *Adrb2^−/−^* mice and BM chimeras were selectively associated with stronger resistance to MCMV (Fig. 1 B, Fig. 2 A, Fig. 4, A and B; and Fig. 5, A and B). The levels of the other inflammatory cytokines/chemokines (CXCL1, IL-10, IL-6, and TNF-α) for which increases were observed in *Adrb2^−/−^* mice (Fig. 2 A) were not regulated by the same mechanisms and were not associated with different survival rates in BM chimeras (Fig. 1 B, Fig. 2 A, Fig. 4 A, and Fig. 5, A and B).

These results suggest that the control of IFN-γ levels by β2-AR signals may affect host survival in this model. As liver NK cells were the main source of this increase in IFN-γ production in *Adrb2^−/−^* mice (Fig. 3, C and D and Fig. S3 C), we investigated the possibility of NK cells being responsible for this greater resistance to infection. *Adrb2^−/−^* mice were treated with an NK1.1-depleting mAb 2 d before MCMV infection (Fig. 5 C). 2 d after anti-NK1.1 mAb treatment, the frequency of NK cells had decreased by 93%, 76%, and 88% in the blood, spleen, and liver, respectively. Anti-NK1.1 mAb treatment greatly decreased the resistance of *Adrb2^−/−^* mice to MCMV, as the survival of NK cell–depleted *Adrb2^−/−^* mice (40%) was lower than that of NK cell–sufficient *Adrb2^−/−^* mice (70%; Fig. 5 C). However, NK cells were not the only cell type contributing to the higher resistance of *Adrb2^−/−^* mice to infection, as NK cell–depleted control (*Adrb2^+/+^*) animals remained more susceptible to MCMV than did NK cell–depleted *Adrb2^−/−^* mice (Fig. 5 C). The frequency of ILC1 in the liver also decreased by 96% upon NK1.1 depletion. We cannot, therefore, rule out a role for this cell type in the phenotype observed upon anti-NK1.1 mAb treatment. However, the observation of a specific increase in the number of IFN-γ–producing liver NK cells but not ILC1s in infected *Adrb2^−/−^* mice relative to their control littermates (Fig. 3) favors a model in which NK cells play a major role.

### β2-AR signals controlling IFN-γ production and host susceptibility to MCMV are not induced through catecholaminergic innervation

We investigated whether the host response to MCMV was controlled by regulation of the β2-AR pathway by noradrenergic neurons in tissue or systemically by blood catecholamine levels. Mice were systemically treated with the catecholaminergic neurotoxin 6-hydroxydopamine (6-OHDA) for this purpose (Fig. 5 D). 6-OHDA treatment is used to selectively target noradrenergic neurons without affecting the adrenal medulla or plasma adrenaline levels (Clark et al., 1972; Tsunokuma et al., 2017). As expected, 6-OHDA treatment induced the ablation of tyrosine hydroxylase (TH)^+^ sympathetic fibers in both the spleen and the liver (Fig. 5 E; data not shown). Control (PBS-treated) and 6-OHDA–treated mice then received injections of anti-NK1.1 antibody or an isotypic control and were infected with MCMV (Fig. 5 D). In mice treated with the isotype control antibody, sympathetic denervation did not affect IFN-γ levels in the bloodstream 44 h after MCMV infection (Fig. 5 F). By contrast, NK1.1 depletion induced a large decrease in IFN-γ levels, confirming that NK1.1^+^ cells are the main source of systemic IFN-γ at this time point (Fig. 5 F). Thus, catecholaminergic neurons are not involved in the regulation of IFN-γ production by NK1.1^+^ cells in MCMV-infected mice.

Consistent with a major role of IFN-γ in this model, sympathetic denervation did not affect survival in mice treated with the isotype control antibody (Fig. 5 G, black lines). Moreover, NK1.1^+^ cell depletion increased susceptibility to infection similarly in PBS-treated and 6-OHDA–treated mice (Fig. 5 G, purple lines). Thus, sympathetic innervation does not contribute to the increased host resistance to MCMV infection observed in *Adrb2^−/−^* mice.

Collectively, these data support a model (Fig. 5 H) in which catecholamines produced by the adrenal gland and released systemically in the bloodstream act on nonhematopoietic cells in tissues via β2-AR to modulate proinflammatory signals. These proinflammatory signals are important for NK cell activation, and their modulation affects the NK cell IFN-γ response, which is necessary for efficient viral clearance. The modulation of the systemic IFN-γ response by this adrenergic pathway reduces the control of viral replication and increases the severity of tissue lesions, especially in the spleen, decreasing host resistance to infection.

### Concluding remarks

This study highlights the mechanisms by which the stress pathway can increase host susceptibility to viral infection. Stimulation of the β2-AR pathway was found to be detrimental for host survival to MCMV infection, suggesting that the stress mediators adrenaline and noradrenaline have a negative impact on host resistance to infection. Consistent with this hypothesis, β2-AR deficiency resulted in a higher resistance to infection, which was associated with stronger IFN-γ responses in liver NK cells. This stronger response in β2-AR–deficient mice was associated with a better control of viral replication and less severe tissue damage. Moreover, NK cell depletion reduced the survival of β2-AR–deficient mice. However, the regulation of IFN-γ production in liver NK cells was not cell intrinsic and involved β2-AR expression in radio-resistant nonhematopoietic cells. These results are consistent with previous studies showing that host susceptibility to infection involves, not only the host immune system, but also the ability of parenchymal tissues to tolerate or to react to pathogen-induced dysfunctions (Medzhitov et al., 2012; Soares et al., 2017). Further studies are required to investigate in greater detail the contribution of β2-AR in nonhematopoietic cell types, particularly in the liver.

The role of β2-AR appears to be different at different stages of MCMV infection. Indeed, we show that this pathway is a cell-extrinsic negative regulator of NK cell IFN-γ production at early stages of infection, with a cost in terms of host resistance. By contrast, at later stages, cell-intrinsic adrenergic signaling can be protective and promotes the adaptive response and expansion of the NK cell population in the spleen (Diaz-Salazar et al., 2020). This secondary level of regulation may account, at least in part, for the maintenance of this pathway during evolution.

Interestingly, Diaz-Salazar et al. (2020) observed a modest intrinsic role of β2-AR in modulating early IFN-γ production upon MCMV infection. This role was revealed in the context of mixed-BM chimera experiments in which β2-AR–deficient NK cells are in competition with WT NK cells for their development and activation. This phenotype was associated with a defect in the maturation status of β2-AR–deficient NK cells compared with β2-AR–sufficient NK cells present in the same recipient. Such functional and maturation defects were not observed in *Adrb2^Ncr1iCre^* mice (Fig. 3 G; Diaz-Salazar et al., 2020), suggesting that the role of β2-AR signaling in NK cells is context dependent. Consistent with this hypothesis, the β2-AR can be coupled to different intracellular pathways depending on its state when activated, inducing different intracellular responses upon ligand binding (Matera et al., 2018). For example, the engagement of the β2-AR on T and B lymphocytes regulates their function according to the molecular signaling pathway activated, the cytokine microenvironment, and the time of receptor engagement in relation to the activation and differentiation state of the cell (Sanders, 2012). This complexity may explain some of the controversies in the literature suggesting apparently conflicting functions of β2-ARs in immune cells (Sanders, 2012; Wu et al., 2018).

Clinical studies revealed that psychological stress is associated with a higher risk of developing acute infectious illness (Cohen et al., 1991; Glaser and Kiecolt-Glaser, 2005; Irwin and Cole, 2011). It will be important to determine whether these effects are at least partly mediated by β2-AR signals and to determine whether β-blocker treatment might be beneficial in some circumstances.

This study expands our understanding of host protection from infectious diseases by showing that the stress pathway, by triggering β2-AR signals, downregulates the innate inflammatory response, affecting host fitness.

## Materials and methods

### Mice

C57BL/6J Ly5.2 mice were purchased from Janvier Labs; C57BL/6J Ly5.1 mice were purchased from Charles River; and *Adrb2^LoxP/LoxP^* mice and *Adrb2^−/−^* mice (Hinoi et al., 2008; Chruscinski et al., 1999) were kindly provided by Nicolas Glaichenhaus (Institut de Pharmacologie Moléculaire et Cellulaire, Nice, France). *Ncr1^iCre^* mice (Narni-Mancinelli et al., 2011) were obtained from Eric Vivier (Centre d’Immunologie de Marseille Luminy, Marseille, France). *LysM^Cre^* mice (Clausen et al., 1999) were kindly provided by Toby Lawrence (Centre d’Immunologie de Marseille Luminy, Marseille, France). All the mice were bred and maintained under specific pathogen–free conditions at the Centre d’Immunophenomique in Marseille and the Centre d’Immunologie de Marseille Luminy. Mice were housed under a standard 12 h/12 h light-dark cycle with food and water ad libitum. Age matched (8–12-wk-old) female mice were used. All experiments were conducted in accordance with institutional committee recommendations (Comité d’Ethique de Marseille no. 14-APAFiS; no. 14260) and French and European guidelines for animal care.

### Clenbuterol treatment

Clenbuterol (catalog no. C5423; Sigma-Aldrich) was added at 9 µg/ml in the drinking water of mice for 7 d before and during MCMV infection. Controls were kept on water alone.

### Organ preparation

Blood was taken through the retro-orbital sinus. The mice were then euthanized, perfused with 10–20 ml PBS 1×, and the spleen and liver were taken. The spleen and liver were smashed through 70-µm cell strainers. Red blood cell lysis was performed with the Red Blood Cell Lysis Buffer from eBioscience on spleen suspensions. Liver lymphocytes were isolated on a 37.5%–67.5% Percoll gradient. Blood was used for Trucount (BD Biosciences) according to the manufacturer’s protocol.

### Flow cytometry

Single-cell suspensions were incubated with the Fc blocking antibody (2.4G2) and with fixable blue dead cell stain kit (Invitrogen). To stain surface molecules the following antibodies were used: anti-CD3 (145-2C11), anti-CD11b (M1/70), anti-CD19 (1D3), anti-CD45.1 (A20), anti-CD45.2 (104), anti-CD49a (Ha31/8), anti-Ly6C (AL-21), anti-Ly6C/Ly6G (RB6-8C5), anti-Ly49H (3D10), anti-MHCII (M5/114.15.2), anti-NK1.1 (PK136), and anti-TCRβ (H57-597) from BD Biosciences; anti-NKp46 (29A1.4), anti-CD49b (DX5 or HMa2), anti-F4/80 (BM8), and anti-Ly49H (3D10) from eBioscience; and anti-CD11c (N418) and anti-Ly6G (1A8) from Biolegend. For intracellular staining, the cells were fixed and permeabilized with an intracellular staining kit (eBioscience), and the following antibodies were used: anti-IFN-γ (XMG1.2) from Biolegend, anti-Ki67 (B56) from BD Biosciences, and anti-GzB (GB12) from Life Technologies. Analysis was performed with FlowJo Software.

### Cytokine analysis

For serum, blood was collected from the retro-orbital sinus of MCMV-infected mice under low stress conditions (i.e., within 2 min of handling). The concentration of IL-6, IL-10, IL-12p70, MIP-1α, MCP-1, KC, TNF-α, and IFN-γ were assessed by cytometric bead array according to the manufacturer’s protocol (BD Biosciences).

### MCMV infection

MCMV (Smith strain) was diluted in DMEM and injected intraperitoneally in female mice at the LD_50_. DMEM only was injected in the uninfected control group. All MCMV injections were done between 2 pm and 4 pm. For survival experiments, the mice were weighed every 24 h and checked for signs of distress. For the time point experiments, blood was taken via the retro-orbital sinus, mice were euthanized and perfused, and organs were harvested for further analysis. For the NK cell depletion experiments, 100 µg of anti-NK1.1 (PK136) from bioXcell or vehicle were injected via the retro-orbital sinus 2 d before MCMV infection.

### In vitro NK cell activation

Splenocyte suspensions were distributed in a 96-well 2HB Immulon plate precoated with antibody against NK1.1 (PK136; 27 µg/ml) in the presence of noradrenaline (10 µM) or diluent (control). Cells were activated in the presence of monensin (GolgiStop; BD Biosciences) in complete medium RPMI 1640 (Gibco/Invitrogen) supplemented with 10% fetal calf serum, 1 mM sodium pyruvate, 10 mM Hepes, penicillin (100 U/ml), and streptomycin (100 mg/ml). After 4 h at 37°C, cell-surface staining was performed. For intracellular IFN-γ staining, cells were fixed with 2% paraformaldehyde and permeabilized with Perm/Wash solution (BD PharMingen).

### Viral titer and quantitative real-time PCR

Organs were kept in RNAlater (Qiagen) after harvesting. RNA was extracted from organ homogenates with the RNeasy Fibrous Tissue Mini Kit (Qiagen) and reverse transcribed with the iScript cDNA Synthesis kit (Bio-Rad Laboratories). Viral titers were determined, by quantitative PCR, as absolute levels of the Ie1 gene (forward: 5′-GAG​TCT​GGA​ACC​GAA​ACC​GT-3′; reverse: 5′-GTC​GCT​GTT​ATC​ATT​CCC​CAC-3′; Sigma-Aldrich) using the SYBR Green Master Mix (Takara).

### Histology

For immunofluorescence staining, tissues were fixed in Antigenfix (Diapath) for 3–4 h, dehydrated in 30% sucrose overnight at 4°C, and embedded in TissueTek optimal cutting temperature compound (Sakura). Sections of 8 µm were cut using a Cryostat Leica 3050s and mounted on slides. The sections were rehydrated with PBS, blocked with 2% BSA, permeabilized with 0.3% X100-Triton, and stained with anti-CD31 (553370; BD PharMingen) and anti-TH (AB152; Millipore) antibodies overnight at 4°C. For histological analysis, tissues were fixed in 10% neutral buffered formalin, dehydrated, and embedded in paraffin. Sections of 3.5 µm were cut using the microtome Leica RM2245. H&E staining was effectuated automatically with Leica autostainer XL, and slides were mounted with Entellan and kept at room temperature. Histological slides of spleen and liver tissue were assessed by an anatomopathologist in a blinded way. For spleen inflammation grading, a score was assigned based on the severity: 0 for a normal spleen, 1 for mild (multifocal pyogranulomas in marginal zones), 2 for moderate (locally coalescing pyogranulomas in marginal zones with small necrotic foci), 3 for marked (large and coalescing pyogranulomas throughout the splenic parenchyma with extensive necrotic foci, the periarteriolar lymphoid sheaths are preserved), and 4 for severe (extensive necrotic and pyogranulomatous foci; periarteriolar lymphoid sheaths are partially replaced by necrotic and granulomatous inflammation). For liver inflammation grading, a score was assigned based on the severity: 0 for normal, 1 for mild (multifocal pyogranulomatous hepatitis with scattered single necrotic hepatocytes), 2 for moderate (multifocal to coalescing necrotic and pyogranulomatous hepatitis with intranuclear inclusions in hepatocytes), and 3 for marked (coalescing necrotic and pyogranulomatous hepatitis with intranuclear inclusions in hepatocytes). Analysis was performed on random fields, chosen on digitally scanned spleen and liver sections (Case Viewer Software, 3Dhistech).

### ALT activity assay

An ALT activity assay was done on serum samples with the ALT Activity Assay Kit from Sigma-Aldrich (I0634).

### Generation of BM chimeras

Before treatment, donor and recipient mice were kept on Bactrim in the drinking water for 1 wk. Recipient mice at the age of 6–7 wk were irradiated once with 5.5 Gy. 1 d later, 5 × 10^6^ BM cells of donor mice were transferred via injection in the retro-orbital sinus. Mice were then kept on Bactrim in the drinking water for ≤1 mo after irradiation. Experiments were performed 8–9 wk after BM transfer. All mice showed a chimerism of a minimum of 85% in the liver and spleen (data not shown).

### Statistical analysis

Statistical analysis was achieved with GraphPad Prism Software. Data were considered statistically significant when the P value was < 0.05 (*, P < 0.05; **, P < 0.01). Data were compared by an unpaired Student’s *t* test when values followed a Gaussian distribution with similar variances or with the Mann-Whitney *U* test. For multigroup comparisons, we applied one-way ANOVA or multiple *t* test. Differences in survival were evaluated with the Mantel-Cox test.

### Online supplemental material

Fig. S1 shows that viral clearance and tissue damage in the liver of *Adrb2^−/−^* mice after MCMV infection is unaffected compared with their littermate controls. Fig. S2 shows that β2-AR deficiency does not alter the trafficking of major immune cell subsets upon MCMV infection. Fig. S3 shows the inflammatory cytokine and innate immune responses in MCMV infected *Adrb2^−/−^* and *Adrb2^LysMCre^* mice.
